# Freezing of gait in idiopathic normal pressure hydrocephalus

**DOI:** 10.1186/s12987-024-00522-y

**Published:** 2024-03-08

**Authors:** Carl-Johan Kihlstedt, Jan Malm, Alfonso Fasano, David Bäckström

**Affiliations:** 1 Department of Clinical Science, Neurosciences, Umeå University, Umeå, Sweden; 2 Edmond J. Safra Program in Parkinson’s Disease, Morton and Gloria Shulman Movement Disorders Clinic, Toronto Western Hospital, UHN, Toronto, ON Canada; 3 Division of Neurology, University of Toronto, Toronto, ON Canada; 4 Krembil Brain Institute, Toronto, ON Canada; 5 Center for Advancing Neurotechnological Innovation to Application (CRANIA), Toronto, ON Canada

**Keywords:** Hydrocephalus, normal pressure, Gait disorders, Neurologic, Parkinson disease, Parkinsonian disorders, Cerebrospinal fluid shunts

## Abstract

**Background:**

Reports of freezing of gait (FoG) in idiopathic normal pressure hydrocephalus (iNPH) are few and results are variable. This study’s objective was to evaluate the frequency of FoG in a large cohort of iNPH patients, identify FoG-associated factors, and assess FoG’s responsiveness to shunt surgery.

**Methods:**

Videotaped standardized gait protocols with iNPH patients pre- and post-shunt surgery (*n* = 139; median age 75 (71–79) years; 48 women) were evaluated for FoG episodes by two observers (Cohens kappa = 0.9, *p* < 0.001). FoG episodes were categorized. Mini-mental state examination (MMSE) and MRI white matter hyperintensities (WMH) assessment using the Fazekas scale were performed. CSF was analyzed for Beta-amyloid, Tau, and Phospho-tau. Patients with and without FoG were compared.

**Results:**

Twenty-two patients (16%) displayed FoG at baseline, decreasing to seven (8%) after CSF shunt surgery (*p* = 0.039). The symptom was most frequently exhibited during turning (*n* = 16, 73%). Patients displaying FoG were older (77.5 vs. 74.6 years; *p* = 0.029), had a slower walking speed (0.59 vs. 0.89 m/s; *p* < 0.001), a lower Tinetti POMA score (6.8 vs. 10.8; *p* < 0.001), lower MMSE score (21.3 vs. 24.0; *p* = 0.031), and longer disease duration (4.2 vs. 2.3 years; *p* < 0.001) compared to patients not displaying FoG. WMH or CSF biomarkers did not differ between the groups.

**Conclusions:**

FoG is occurring frequently in iNPH patients and may be considered a typical feature of iNPH. FoG in iNPH was associated with higher age, longer disease duration, worse cognitive function, and a more unstable gait. Shunt surgery seems to improve the symptom.

## Background

Freezing of gait (FoG) is a disabling motor symptom defined as a “*brief episodic absence or marked reduction of forward progression of the feet despite the intention to walk*” [1]. Reports of freezing of gait (FoG) in idiopathic normal pressure hydrocephalus (iNPH) are few and results are variable (Table 1), in contrast to Parkinson’s disease (PD) where FoG is routinely reported, and the overall prevalence reaches higher than 50% [2]. FoG also appears in other parkinsonian syndromes and after stroke [3, 4]. 

**Table 1:** Summary of studies that report on the frequency of FoG in iNPH patients

Study	Year	Number of participants assessed for FoG	Participants with FoG (%)	Methodology	Video assessment Y/N	Comment
Giladi et al. [4]	1997	37	56	Retrospective clinical series	N	
Stolze et al. [50]	2000	10	30	Case-control study	N	
Tisell et al. [36]	2003	39	42	Case-control study	N	Symptom named “Gait arrest”. 14% post-surgery.
Miyoshi et al. [51]	2005	17	24	Clinical evaluation	N	
Bugalho et al. [52]	2007	15	40	Prospective clinical series	N	
Bugalho et al. [10]	2013	35	20	Case-control study	N	
Souza et al. [27]	2018	25	4	Retrospective clinical series	Y	Does not disclose inter-rater reliability
Agerskov et al. [24]	2018	365	30	Retrospective clinical series	N	Only includes patients who underwent shunt surgery. 8% post-surgery.
Giannini et al. [53]	2019	76	21	Prospective clinical series	N	
Yamada et al. [28]	2021	97	38	Case-control study	Y	7% with probable FoG. Does not disclose inter-rater reliability.

Abbreviations: FoG = Freezing of gait, iNPH = idiopathic normal pressure hydrocephalus

Previous studies documenting FoG in iNPH have reported a high variability in the frequency, with FoG reported in 4–54% of patients (Table 1). Video recordings, i.e., the standard criterion for evaluating FoG, were used by a minority of these studies and none disclosed inter-rater reliability (Table 1). Thus, the frequency of FoG and its impact in iNPH has not been clarified. The symptom was not included in the international iNPH guidelines and is only briefly mentioned in the Japanese iNPH guidelines [5, 6].

FoG further complicates the short-stepped and unstable gait of iNPH patients and may increase the risk of falling, yet it is poorly recognized in this population. FoG is also of particular interest because of its negative effect on the patient’s quality of life (QoL), with an impact even in the early stages of PD [7]. It has been shown that iNPH patients have lower QoL compared to controls [8], therefore it is conceivable that FoG, as in PD, may worsen QoL in iNPH as well.

A link between parkinsonism and iNPH has been suggested. Motor signs (notably the gait pattern), and functional neuroimaging indicate that parkinsonism and nigrostriatal involvement might be present in iNPH patients [9–12]. Further, nigrostriatal deficiency seems to improve after shunt surgery [13]. In this study, we hypothesize that a portion of iNPH patients will present with FoG and that shunt surgery might alleviate this disabling motor phenomenon.

In the present study, we evaluated pre- and postoperative video recordings of 139 iNPH patients. Study objectives were: (1) to determine the frequency of FoG in a large cohort of iNPH patients; (2) to evaluate the effect of shunt surgery on FoG; and (3) to identify factors (including cerebrovascular disease and cerebrospinal fluid– CSF– biomarkers) associated with FoG.

## Methods

In summary, preoperative video recordings of 139 patients with iNPH were assessed regarding the frequency and characteristics of FoG. Clinical features of patients displaying FoG were compared with those who did not. Ninety-one of the patients underwent ventriculo-peritoneal-shunt surgery with an adjustable STRATA® valve (Medtronic, Minneapolis, MN, US) and were further screened for frequency of postoperative FoG on their video recordings at the time of valve setting optimization (median follow-up duration: 168 (112–247) days).

### Study population

Patients in northern Sweden who presented with ventriculomegaly and clinical symptoms of hydrocephalus were referred to and investigated at the neurological department in Umea. All patients were examined using a standardized protocol that included Magnetic Resonance Imaging (MRI), standardized video recording of balance and gait, large volume lumbar puncture (tap test), CSF infusion test, and blood screening. In uncertain cases, external lumbar drainage was also performed.

The study population in the present study consisted of 139 iNPH patients (median age 75 (71–79) years; 48 women). Clinical features are described in Table 2. The 139 participants were randomly selected from 377 patients with iNPH examined between 2007 and 2019. The diagnosis was based on patient history and neurological and radiological examination as described in the international iNPH guidelines [5]. The patients were categorized as having “probable” iNPH i.e., normal intracranial pressure (ICP), neuroimaging with ventriculomegaly (Evans index > 0.3), and gait/ balance disturbance as well as either cognitive impairment or urinary incontinence/ urgency.

**Table 2:** Patient characteristics at baseline, comparing those who displayed at least one FoG episode with those who did not, pre-surgery

	Total sample (*n* = 139)	Patients without FoG (*n* = 117)	Patients with FoG (*n* = 22)	*p*-value
**Age**	**75 (71–79)**	**75 (71–78)**	**78 (76–79)**	**0.029** ^***a***^
Female sex	48 (35%)	41 (35%)	7 (32%)	0.77^*b*^
Smoker	16 (12%) (*n* = 136)	14 (12%) (*n* = 114)	2 (9%)	1.0^*d*^
Diabetes	52 (37%)	40 (34%)	12 (55%)	0.07^*b*^
Parkinsonian syndrome	7 (5.0%)	6 (5.1%)	1 (4.5%)	1.*0*^*d*^
MMSE*	**25 (21–27)** **(** *n* **= 138)**	**25 (22–27)** **(** *n* **= 116)**	**21 (17–26)**	**0.** ***031*** ^***c***^
**Disease duration (years)**	**2 (1–3)** **(** *n* **= 131)**	**2 (1–3)** **(** *n* **= 109)**	**4 (2–5)**	**< 0.001** ^**c**^
**Maximum walking speed (m/s)**	**0.83 (0.60–1.10)** **(** *n* **= 137)**	**0.87 (0.68–1.10)** **(** *n* **= 116)**	**0.57 (0.45–0.73)** **(** *n* **= 21)**	**< 0.001** ^**a**^
Tinetti POMA balance score*	**11 (8–13)**	**11 (9–14)**	**7 (4–10)**	**< 0.001** ^**c**^
Tinetti POMA gait score*	**8 (5–10)**	**8 (6–10)**	**3 (1–5)**	**< 0.001** ^**c**^
Tinetti POMA test total score*	**18.0 (13.0–22.0)**	**20.0 (15.0–23.0)**	**9.5 (6.0–14.0)**	**< 0.001** ^**c**^
TUG Time (seconds)	18.1 (14.3–25.1) (*n* = 74)	18.1 (14.3–24.0) (*n* = 69)	27.5 (15.3–28.0) (*n* = 5)	0.489^c^
Shunt surgery	91 (66%)	76 (65%)	15 (68%)	0.77^b^
Median follow-up-time (days)	168 (112–247) (*n* = 91)	162 (110–244) (*n* = 76)	215 (122–265) (*n* = 15)	0.33^c^
Fazekas grade	2 (2–3) (*n* = 123)	2 (2–3) (*n* = 106)	2 (1–2) (*n* = 17)	0.243^c^
Framingham Risk Score	30.7 (20.2–44.5) (*n* = 132)	29.6 (20.0-43.2) (*n* = 110)	36.5 (21.2–48.9)	0.323^c^

Values indicate median (interquartile range) or no. (%). *P*-value representing significance in the difference between patients with and without FoG for each variable. Sample sizes are displayed in the top row unless stated otherwise in a specific cell. Statistically, significant comparisons are bold-typed

Abbreviations: * Higher is better, ^a^= Independent two-tailed t-test, ^b^= Person chi-square, ^c^= Independent-samples Mann-Whitney U test, ^d^= Fisher’s exact test, FoG = Freezing of gait, n = sample size, no.=number of patients, TUG = Time up and go, SD = Standard deviation, POMA = Performance Oriented Mobility Assessment

### Video gait assessment

The pre- and postoperative study protocol included video recordings of patients performed by a dedicated physiotherapist. Two video sequences were used to screen for FoG: The first video sequence was the gait part of the “Tinetti Performance Oriented Mobility Assessment (POMA)” test [14]. In this test, the patients walked normally across the room, turned around (freely choosing direction), and walked back. First, they walked two times with the camera recording them from the side and then two times facing the camera. The second video sequence was the “Timed up and go (TUG)” test [15]. Patients rose from a chair without armrests and walked three meters straight as fast as possible, walked back, and sat down. The camera was filming them from the side. The TUG test was introduced to the investigation scheme in 2013 and was thus not part of all video recordings.

The pre- and postoperative video recordings were evaluated for FoG by two trained investigators. One is a neurologist specialized in movement disorders who reviewed a portion of the video recordings to ensure an estimation of the inter-rater reliability (see below). A FoG episode was defined as an “*Unsuccessful attempt to initiate or continue locomotion or a transient and clinically distinctive break in locomotion for no apparent reason*” [1]. FoG episodes were further classified after freezing characteristics [16, 17]: (1) markedly smaller steps/shuffling with minimal forward motion, (2) trembling in place with no forward motion, or (3) total akinesia. In cases with mixed episodes, only one value was recorded using the hierarchy 3 > 2 > 1. Situations triggering FoG were classified as: (a) start hesitation (unable to initiate gait), (b) turning, (c) straight walking.

### Additional assessment

Maximum gait velocity was measured by a dedicated physiotherapist. The patients walked at their maximum speed for ten meters, this was repeated six times and the mean was calculated. The patient’s overall gait and balance were also scored by the physiotherapist according to the “Tinetti balance and gait assessment” [14]. The Tinetti score was further used to assess the risk of falls [18]. Cognition was assessed with the Mini-Mental State Examination (MMSE) [19].

White matter hyperintensities on MRI were assessed using the Fazekas scale [20]. Every patient was scored 0–3 points for periventricular white matter (PWM) lesions and deep white matter (DWM) lesions by assessing their MRI transverse FLAIR sequence. Then the sum of PWM and DWM scores were calculated to obtain a total score; 0 points = No hyperintensities, 1–2 = mild, 3–4 = moderate, and 5–6 = severe.

The participant’s cardiovascular risk was assessed using the second version of the Framingham risk score [21, 22]. Patients with overlapping iNPH and PD were recorded. PD diagnosis was based on consensus clinical criteria (i.e. bradykinesia as well as either resting tremor or muscle rigidity and responsiveness to dopaminergic treatment). Beta-amyloid _1−42_, Tau, and Phospho-tau levels in CSF were analyzed using procedures approved by the Swedish Board for Accreditation and Conformity Assessment, blinded to clinical data.

### Inter-rater reliability

The inter-observation reliability for the FoG identification and Fazekas scoring was assessed by calculating Cohen’s kappa coefficient [23]. For this purpose, 20 patients of whom ten were with FoG and ten without FoG were randomly selected and assessed by a movement-specialized neurologist. FoG identification had a “good” level of agreement (Cohens kappa = 0.9, *p* < 0.001). The Fazekas scale scoring had a “moderate” level of agreement (Cohens kappa = 0.762, *p* < 0.001).

### Statistical analysis

Descriptive statistics were calculated. We compared patients with and without FoG based on independent two-sample t-test, Pearson’s chi-squared test, independent samples Mann–Whitney U test, and Fisher’s exact test depending on data distribution and numerosity. Multiple linear regression was used to adjust results for confounders. The McNemar test was used to compare patients with FoG pre- and post-shunt surgery. All data were analyzed using SPSS® version 28.0.1.1 (Armonk, New York, USA).

## Results

### FoG at baseline

**Fig. 1:**
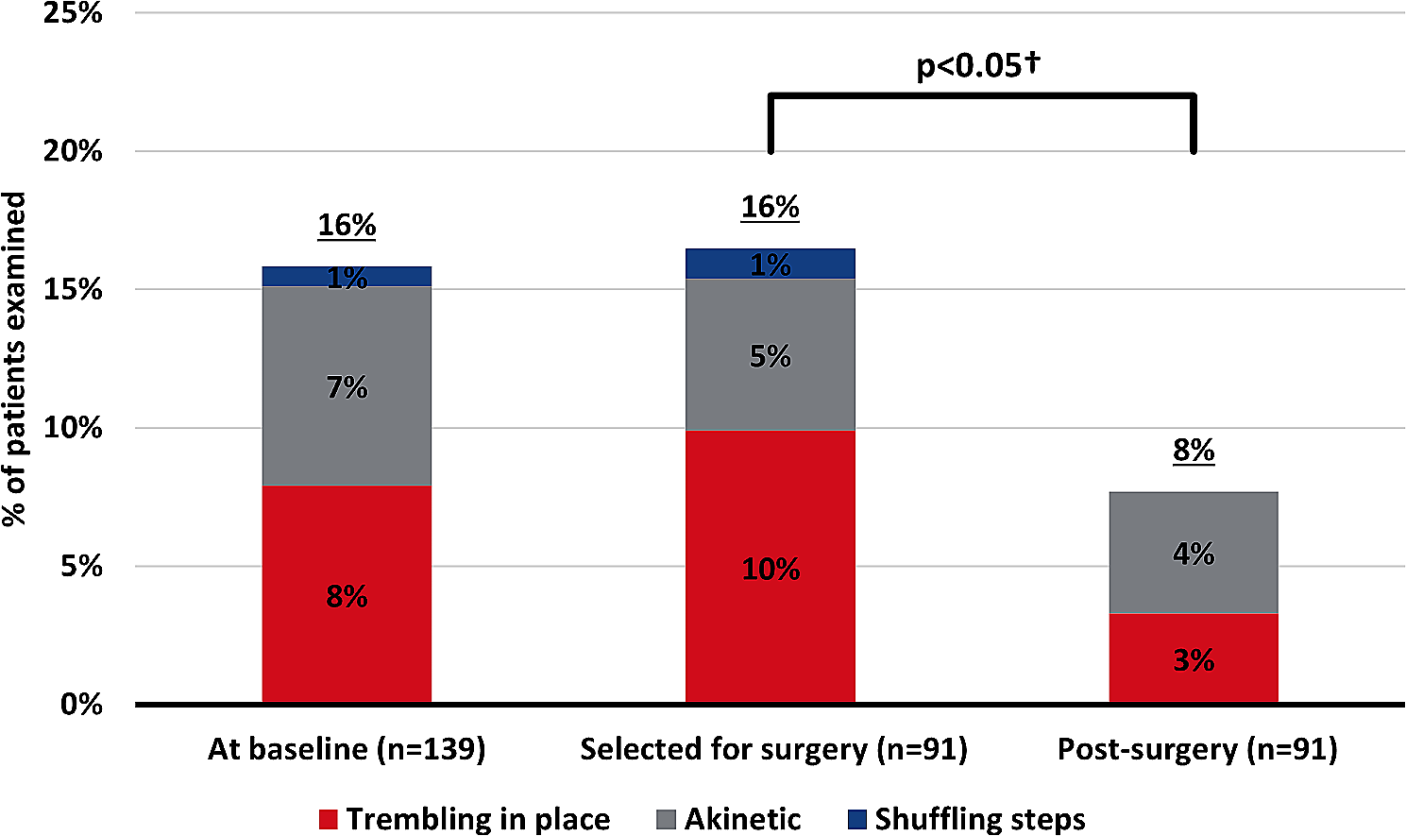
**FoG attributes at baseline, pre- and, post-surgery**. The total frequency of FoG and the distribution of freezing characteristics. Shows the distribution of patients with different FoG freezing characteristics and the total percentage displaying FoG out of the total number of investigated patients with iNPH, at baseline, after selection for surgery, and after surgery. ^**†**^McNemar test

**Fig. 2:**
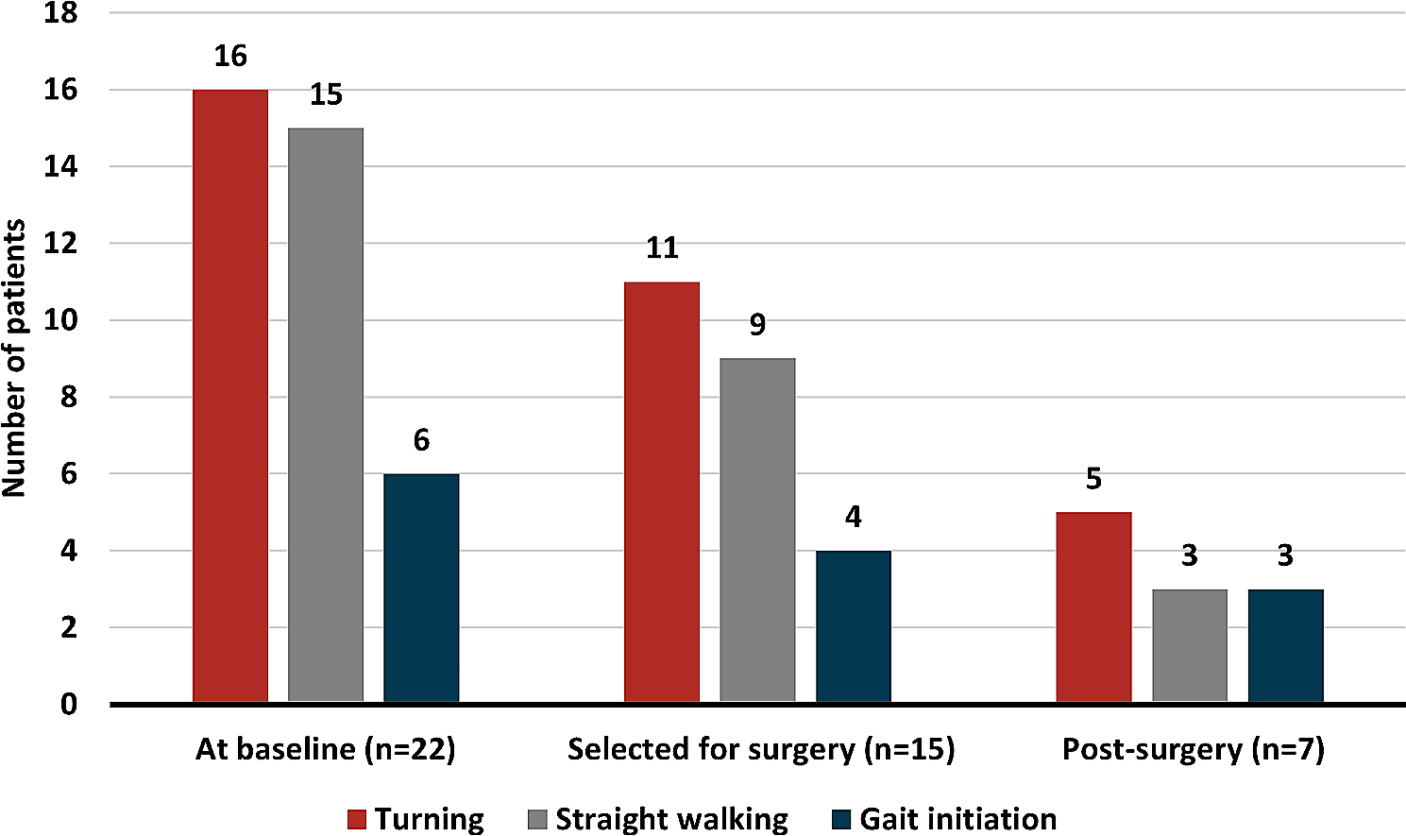
**The number of patients who froze in each situation**. Shows the number of iNPH patients with FoG who froze in each situation, at baseline, after selection for surgery, and after surgery. Note that, as shown in 1B, most of the patients with FoG display FoG in more than one situation

Video recordings of 139 patients at baseline revealed 22 patients (16%) with FoG (Fig. 1). FoG was most commonly occurring during turning (73%) or straight walking (68%), while fewer experienced freezes during gait initiation (27%) (Fig. 2). The most frequent freezing characteristic was “trembling in place” (50%), followed by “akinetic” (46%) (Fig. 1). Only one patient had FoG characterized by “shuffling steps”.

Out of the 22 patients with FoG at baseline one patient had overlapping iNPH and parkinsonian syndrome (PD). In the whole sample seven patients had a parkinsonian syndrome, and out of these four had CSF shunt surgeries. PD was diagnosed in five of the patients, with one having FoG at baseline. Lewy-body dementia and multiple system atrophy type *P* were present in one patient each, none with FoG.

### FoG after CSF shunt surgery

Ninety-one patients had CSF shunt surgery, of whom 15 displayed FoG at baseline (Fig. 1). After surgery, two-thirds (*n* = 10 out of 15; 67%) no longer exhibited the symptom (McNemar test: *p* = 0.039). In total seven patients (8%) showed FoG post-surgery, the symptom persisted in five patients, and two patients who were without FoG at baseline now had FoG. The total number of patients with FoG either at baseline or after CSF shunt surgery was 24 (17%).

### FoG positive versus FoG negative

Comparisons between patients with and without FoG at baseline are shown in Table 2. In summary, patients with FoG were older (77.5 vs. 74.6 years; *p* = 0.029), had lower walking speed (0.59 vs. 0.89 m/s; *p* < 0.001), lower Tinetti POMA score (10.0 vs. 18.6 points; *p* < 0.001), lower MMSE (21.3 vs. 24.0 points; *p* = 0.031), as well as a longer disease duration (4.2 vs. 2.3 years; *p* < 0.001). Adjusted for age and sex with multiple linear regression patients with FoG had lower walking speed (*p* < 0.001, 95% CI [-0.435, -0.121]), lower Tinetti POMA score (*p* < 0.001, 95% CI [-10.602, -5.264]), lower MMSE (*p* = 0.048, 95% CI [-4.400, -0.018]), and longer disease duration (*p* < 0.001, 95% CI [1.078, 2.704]). There were no differences between the groups in terms of sex distribution, the occurrence of diabetes, selection for shunt surgery, Framingham risk score, Fazekas score, or CSF biomarkers (Table 3).

**Table 3:** Medians of CSF biomarkers in patients with and without FoG.

	Total sample	Patients without FoG	Patients with FoG	*p*-value
Beta-Amyloid_1 − 42_	419 (323–556) (*n* = 124)	430 (319–562) (*n* = 102)	387 (331–490) (*n* = 22)	0.811^a^
Tau	202 (158–286) (*n* = 125)	202 (158–286) (*n* = 103)	208 (160–302) (*n* = 22)	0.946^b^
Phospho-Tau	30 (24-40) (*n* = 121)	30 (24-40) (*n* = 101)	32 (27-38) (*n* = 20)	0.842^b^

Values indicate median (interquartile range). *P*-value representing significance in the difference between patients with and without FoG for each variable

Abbreviations: ^a^= Independent two-tailed t-test, ^b^=Independent-samples Mann-Whitney U test, FoG = Freezing of gait, n = sample size

## Discussion

In this study, pre- and postoperative video recordings of gait and balance in 139 iNPH patients were studied concerning FoG. 16% had FoG episodes. FoG should be considered as a typical feature of iNPH.

With 16% of patients displaying FoG at baseline, the symptom seems to be an integral but underrecognized feature of the iNPH syndrome. Only a small number of earlier studies with mostly small sample sizes have described FoG in iNPH patients (Table 1). The frequencies reported vary widely. The largest of these studies with 365 patients examined between 1982 and 2016, reported a frequency of 30% pre-surgery [24]. However, the solidity of this finding is uncertain as the study has an unclear definition of FoG, only assesses patients selected for shunt surgery, is retrospective, includes patients over a 30-year period, and does not use video recordings for assessment.

Due to the episodic nature of FoG, the symptom can sometimes be hard to identify and assess. Evaluation of video recordings is a traditional way of streamlining the process of assessing FoG [25, 26]. Two earlier studies have used video recordings for FoG assessment, including 25 and 97 patients respectively, and found a frequency of 4% and 38% [27, 28]. None of these studies disclosed any inter-rater reliability. In the present study, a good agreement between the two examiners was achieved.

Based on a recent review, the prevalence of FoG in PD patients with a disease duration of fewer than five years is only 14% [2]. Our data suggest that FoG in iNPH could be as frequent or more frequent than FoG in the early stages of PD, thus representing a useful clinical sign guiding the differential diagnosis during the early investigation. In contrast with PD [16, 29], the shuffling FoG phenotype seems very uncommon in iNPH. We found no earlier studies describing the characteristics of FoG in iNPH. As for the triggers, FoG in iNPH patients was most common during straight walking or turning, once again an aspect not investigated hitherto.

Turning is a well-known task able to elicit FoG in PD patients [16, 29], it is particularly effective when asking the patient to perform rapid 360-degree turns in both directions. Another well-known elicitor is carrying out a cognitive dual task while performing maneuvers [30]. Both these techniques could also be tried in iNPH patients when searching for FoG, either separately or combined.

In keeping with what is seen in PD, iNPH patients with FoG identified in this study had a slower gait velocity. Notably, none displayed festination (rapid acceleration of shuffling steps without motor blocks), which is reported in PD patients with FoG. Some iNPH patients report this problem at home (personal observation) but it is difficult to capture in the hospital as it usually requires longer walk bouts to become evident. Nonetheless, the lack of festination in patients experiencing FoG may be another trait that differentiates FoG in iNPH and PD.

Patients with FoG identified in this study also had a lower test performance on the Tinetti POMA compared to iNPH patients without FoG. Tinetti POMA scores can be used as a predictor of falls [18], indicating that patients with FoG may have a higher risk of falls compared to patients without FoG. FoG in PD is indeed correlated to an increased risk of falls and increased mortality [31, 32]. Further, the lower Tinetti POMA scores combined with the longer disease duration in patients with FoG might indicate FoG as a late-stage symptom of iNPH, appearing after a progression of motor symptoms.

The FoG association with poorer cognitive function aligns with observations that FoG is often provoked in more cognitively demanding situations such as when dual-tasking (cognitive), turning (motor), or, feeling anxious (limbic) [33]. PD patients with FoG also feature worse cognitive function [34]. Interestingly, PD patients with FoG have reduced acetylcholine in the striatum, temporal, and mesiofrontal limbic regions indicating another possible neuropathological link between FoG and cognition [35].

The video recordings after surgery reveal that FoG occurred less frequently post-surgery compared with pre-surgery, indicating that shunt surgery may ameliorate FoG in iNPH. The total frequency of FoG post-ventriculoperitoneal shunt surgery of 8% corresponds with previous reports of 8–14% [24, 36]. Two patients without preoperative FoG displayed the symptom after surgery. A possibility is that they had the symptom at baseline without showing it during the video-recorded tests. In fact, one of the study limitations is the lack of FoG questionnaires, as this is not part of the standard iNPH assessment. Due to the episodic nature of the symptom, one might use a questionnaire to aid assessment, one example being the “new freezing of gait questionnaire” [37]. However, one should keep in mind that such questionnaires have not been validated in iNPH patients.

The pathophysiology of FoG in iNPH remains speculative. A portion of iNPH patients can present with parkinsonism [9, 38]. Furthermore, a reduction of striatal dopamine reuptake transporters has been shown to correlate with parkinsonism severity in iNPH patients indicating an association [9, 12]. Interestingly, shunt surgery restores some of the dopaminergic innervation [13], thus indicating a mechanical effect of enlarged ventricles on the nigrostriatal fibers. It has been postulated that FoG stems from a disruption of the basal ganglia-supplementary motor area (SMA) loop [1], and several lines of neuroimaging evidence point to an involvement of the caudate nucleus [39]. Accordingly, dopaminergic studies in iNPH patients have also shown the predominant involvement of the caudate nucleus [12], which has a reduced volume in iNPH patients [40]. In addition, the SMA has an increased activity after CSF drainage [41]. This may indicate a progressive impairment of SMA-subcortical connections in untreated patients.

Mechanical effects aside, a minority of iNPH patients might develop PD, as shown by recent studies using alpha-synuclein real-time quaking-induced conversion (RT-QuIC), a highly sensitive and specific technique capable of detecting and amplifying misfolded aggregated forms of α-synuclein in the cerebrospinal fluid [42, 43]. Furthermore, in PD, reduced beta-amyloid levels in CSF correlates with the progression of motor symptoms and the risk of developing FoG [44, 45]. Accordingly, amyloid deposition in the parieto-occipital lobe of PD patients has been linked to FoG occurrence [46], raising the possibility that Alzheimer’s co-pathology plays a role in the pathophysiology of this motor problem in PD. In contrast, amyloid deposition does not seem to contribute to FoG in iNPH, as CSF analysis did not highlight any differences in beta-amyloid, tau, or phospho-tau in patients with and without FoG. This may be another difference between FoG in iNPH compared with PD.

The main strength of this study is the use of standardized video recording for documentation, and that good agreement was found between the two examiners. A further strength is the large number of videos assessed, in total 230.

The main limitation of this study is related to the paroxysmal nature of FoG. Even if a patient does not display FoG during specific tests, it is not certain that the symptom does not occur occasionally. Further, the videotaped tests used lacks some well-known FoG elicitors such as traversing through a narrow space and a cognitive dual task. This means that the actual frequency of FoG could be higher than the one found in this study.

FoG in iNPH patients is a largely uninvestigated and unrecognized issue, only briefly mentioned in the latest Japanese iNPH guidelines and a recent review of iNPH’s clinical features [6, 47]. One study argues that parkinsonism and FoG are routinely observed in iNPH but miscategorized as simply bradykinesia of the limbs with occasional motor blocks [48]. This could partly explain the lack of studies of FoG in iNPH up until now.

Future research should focus on the clinical and epidemiological features of FoG in iNPH– possibly also using home recordings with wearables, the impact FoG has on iNPH patients, and strategies to avoid fall accidents. Attentional and cueing strategies have been modestly effective in overcoming FoG episodes in PD patients at home and could also be tried in iNPH patients [49]. Video assessment should include a gait examination designed to elicit FoG by using various cognitive and physical challenges, and importantly including repetitive turning tasks.

## Conclusions

We have shown that FoG is a common sign of iNPH and needs further investigation. Interestingly, the proportion of iNPH patients displaying FoG was reduced post-shunt surgery. FoG was associated with higher age, worse cognitive function, a slower and more unstable gait as well as a longer disease duration. Clinicians should be attentive of patients with iNPH exhibiting FoG pre- or post-shunt surgery since the identification of FoG may improve fall prevention efforts.

## Data Availability

No datasets were generated or analysed during the current study.

## Abbreviations

FoG: Freezing of Gait
iNPH: Idiopathic normal pressure hydrocephalus
MMSE: Mini-mental state examination
MRI: Magnetic resonance imaging
WMH: White matter hyperintensities
CSF: Cerebrospinal fluid
PD: Parkinson’s disease
QoL: Quality of life
ICP: Intracranial pressure
POMA: Performance Oriented Mobility Assessment
TUG: Timed up and go
PWM: Periventricular white matter
DWM: Deep white matter
SMA: Supplementary motor area
RT-QuIC: Real-time quaking-induced conversion
